# Supplementary material for: Frequency modulation of ERK activation dynamics rewires cell fate
Source: Mol Syst Biol. 2015 Nov 30;11(11):838. doi: 10.15252/msb.20156458 (PMC4670727; doi:10.15252/msb.20156458)
Supplement: Supplementary file 1 — Appendix [file MSB-11-838-s001.pdf]

Appendix

Frequency modulation of ERK activation dynamics rewires cell fate

Hyunryul Ryu[1,2], Minhwan Chung[1], Maciej Dobrzyński[3], Dirk Fey[3], Yannick Blum[4] Sung Sik Lee[5], Matthias Peter[5], Boris N. Kholodenko[3*], Noo Li Jeon[1,2*], Olivier Pertz[4,6*]

[1]School of Mechanical and Aerospace Engineering, Seoul National University, Seoul, 151-742, Republic of Korea

[2]Institute of Advanced Machinery and Design, Seoul National University, Seoul, 151-742, Republic of Korea

[3]System Biology Ireland, University College Dublin, Belfield, Dublin 4, Ireland

[4]Dept. of Biomedicine, University of Basel, Mattenstrasse 28, 4058 Basel, Switzerland

[5]Institute of Biochemistry, Otto-Stern-Weg 3, 8093 Zurich, Switzerland

[6]Present address: Institute of Cell Biology, University of Bern, Baltzerstrasse 4, 3012 Bern, Switzerland

*Correspondence and requests for materials should be addressed to O.P. (email:olivier.pertz@izb.unibe.ch), to N.L.J (email:njeon@snu.ac.kr), or to B.N.K (boris.kholodenko@ucd.ie).

Table of Contents

1. Appendix Figures ……………………………………………………………………3

1.1. Appendix Figure S1. PC-12 EKAR2G and microfluidic circuit control experiments…………3

1.2. Appendix Figure S2. Correlation between peak and 40' ERK activation at the single cell level in response to sustained EGF/NGF stimulation at different GF dosages……………………..5

1.3. Appendix Figure S3. Quantification of ERK activity trajectories heterogeneity in response to a 10' NGF pulse…………………………………………………………………...6

1.4. Appendix Figure S4. Quantification of ERK activity trajectories heterogeneity in response to sustained GF stimulation on a 6-hours time scale…………………………………………7

1.5. Appendix Figure S5. Representative single cell ERK activity trajectories in response to 6-hours multipulse 3' GF stimulation regimes at different frequencies………………………...8

1.6. Appendix Figure S6. Quantification of ERK activity trajectories heterogeneity in response to a ensemble-modeled 10' NGF pulse……………………………………………………9

1.7. Appendix Figure S7. Simulations of a model variant where the positive feedback depends on receptor activity according to the Hill equation with coefficient 2 and the threshold K50 = 0.25…………………………………………………………………………..10

1.8. Appendix Figure S8. Additional data for differentiation experiments………………………..11

2. Appendix Table S1……………………………………………………………………….12

3. Appendix Note……………………………………………………………………16

4. Appendix References………………………………………………………………..17

Appendix Figures

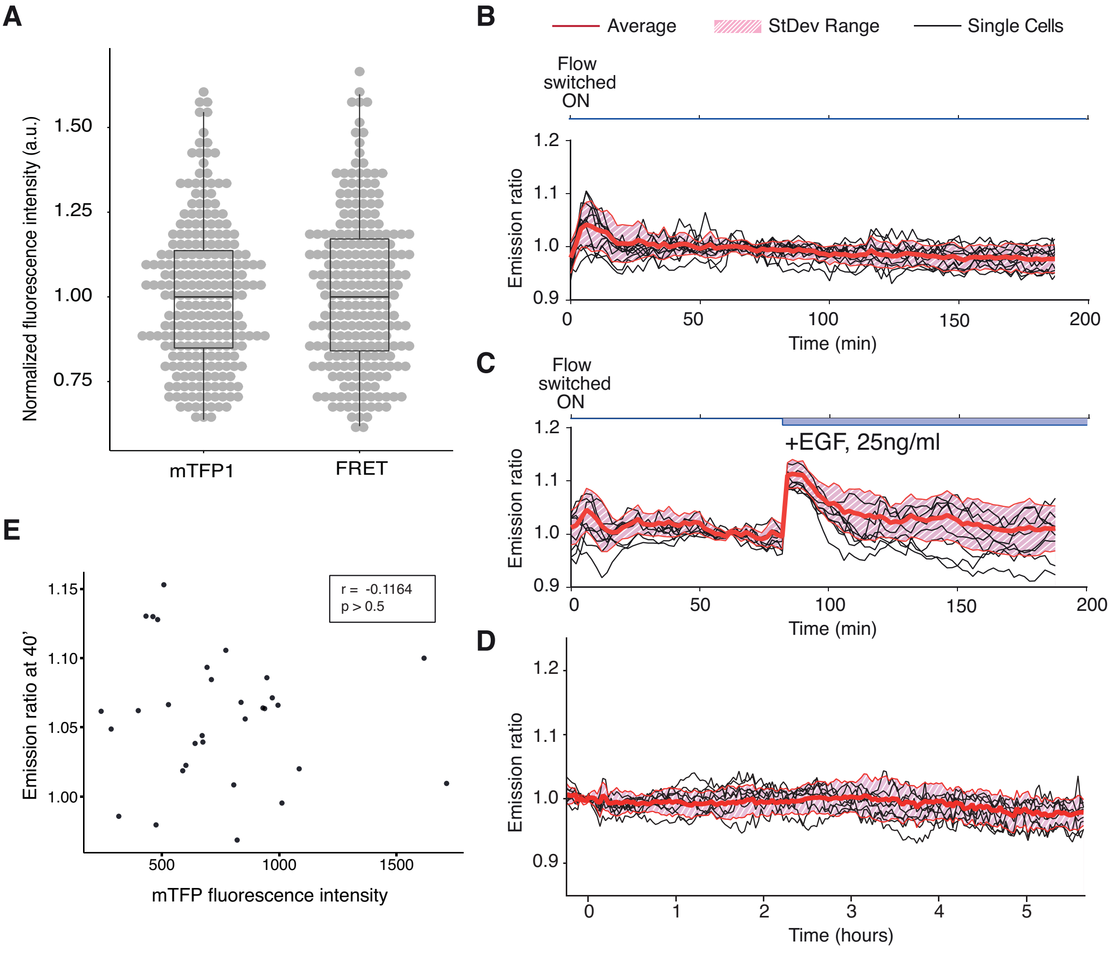

Appendix Figure S1 PC-12 EKAR2G and microfluidic circuit control experiments

A. Measurement of heterogeneity in biosensor expression levels across a population of PC-12/EKAR2G cells. Dotplots of cell-averaged donor (mTFP1) and FRET fluorescence intensities. Median, interquartile range (box) and data within 1.5 IQR range of the lower and upper quartiles (whiskers) are shown. Fluorescence intensity is normalized to the median (n=240 cells).

B-D. ERK activation dynamics baseline in the microfluidic device. Single cell ERK activity trajectories are shown. Cell-averaged ERs, population average and StDev range for n=10 cells. (B) Switching on flow in the microfluidic device leads to induction of an ERK activity transient that subsequently adapts. (C) After adaptation to flow, EGF can trigger an ERK activity transient. (D) After adaptation to flow, ERK activity baseline remains stable for hours. All GF stimulation experiments were therefore started 1 hour after the flow was switched on, a time point at which ERK activity had adapted.

E. Correlation between 40’ ERK activity and EKAR2G expression levels. PC-12 cells were stimulated with 50 ng/ml NGF, which induces a large level of signaling heterogeneity at 45’ post-stimulation. EKAR2G expression levels were approximated by averaging mTFP1 donor channel per cell. For the same, cells the emission ratio at 40’ post-stimulation was calculated. r : Pearson correlation coefficient. P: p-value.

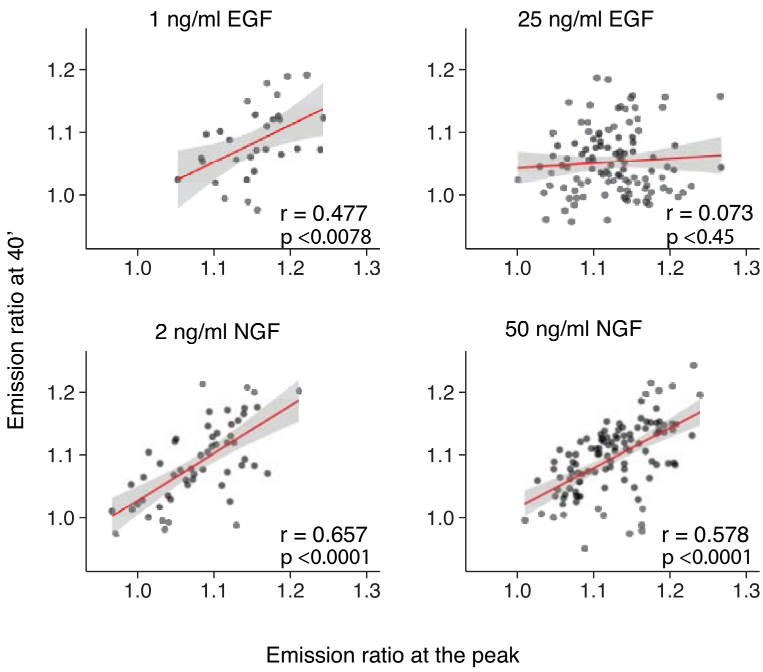

Appendix Figure S2 Correlation between peak and 40' ERK activation at the single cell level in response to sustained EGF/NGF stimulation at different GF dosages.

Plot of peak versus 40' ERK activity emission ratios for single cells. Red line: linear regression, grey region: 95% confidence interval, r : Pearson correlation coefficient.

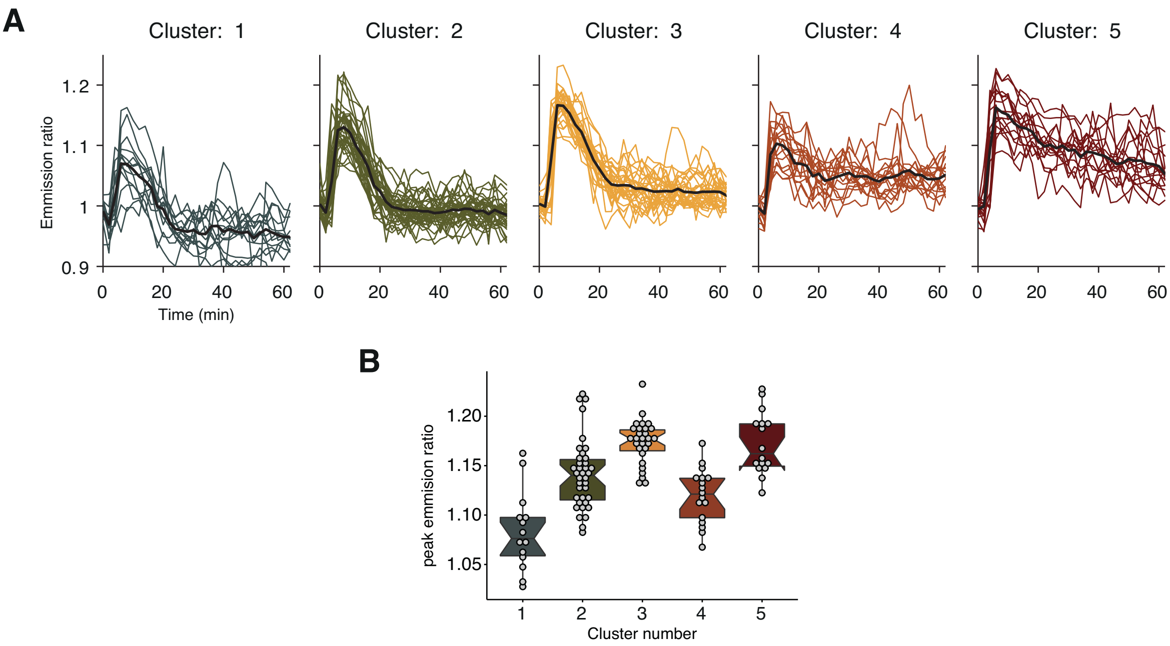

Appendix Figure S3 Quantification of ERK activity trajectories heterogeneity in response to a 10' NGF pulse.

A. Representative ERK activity trajectories identified using k-means clustering. Clusters were sorted according to the mean value of the response within the cluster at 62' after stimulation to differentiate between transient (clusters 1-4) and sustained responses (cluster 5), n = 112 measured cells.

B. Peak emission ratio intensity for each cluster shown in (B). Notched boxplots with median, interquartile (box) and 1.5 IQR (whiskers) range and raw datasets are shown.

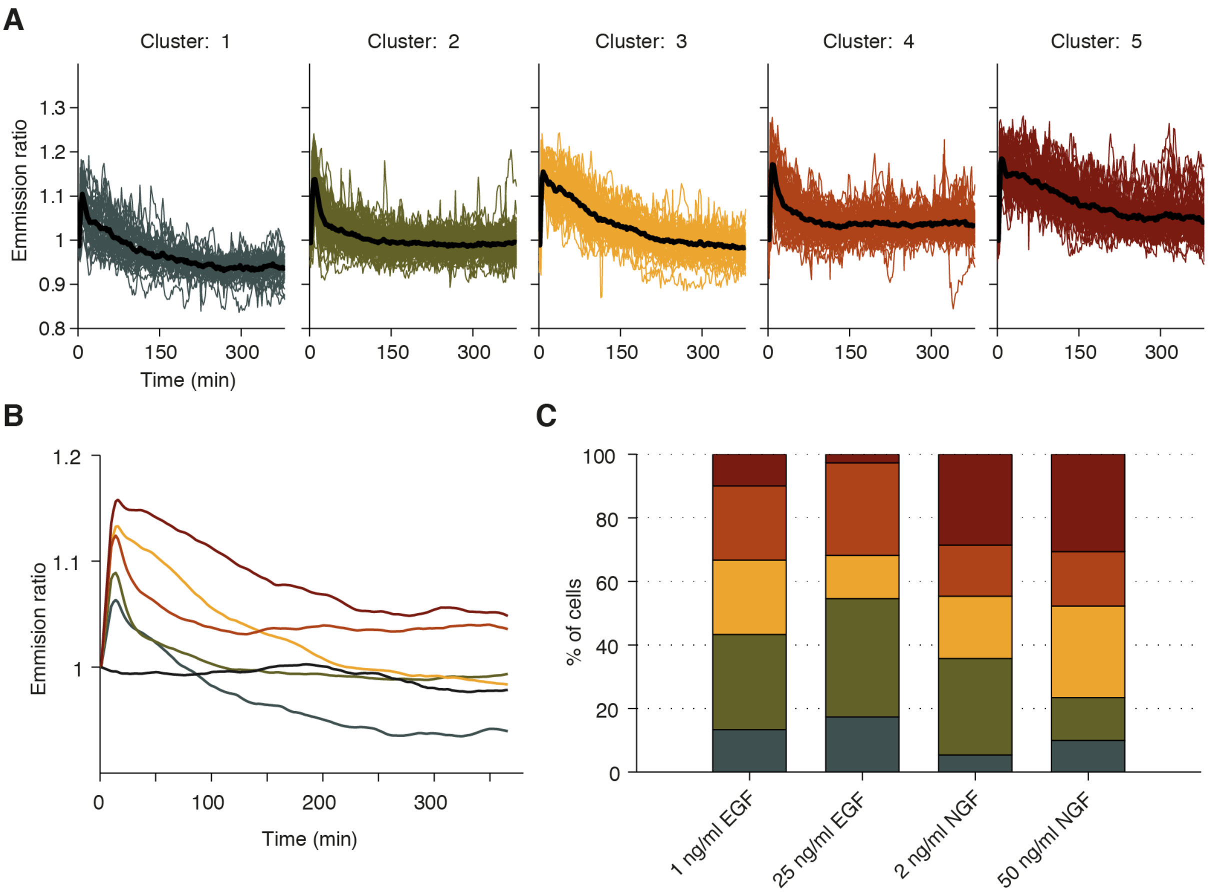

Appendix Figure S4 Quantification of ERK activity trajectories heterogeneity in response to sustained GF stimulation on a 6-hours time scale.

The same dataset as shown in Fig 2A was analyzed by k-means clustering at this longer timescale (n=307 cells).

A. Raw (color-coded by cluster) and cluster representative trajectories (black).

B. Overlayed cluster representative trajectories.

C. Population distribution of representative ERK activity trajectories in response to different GF dosages.

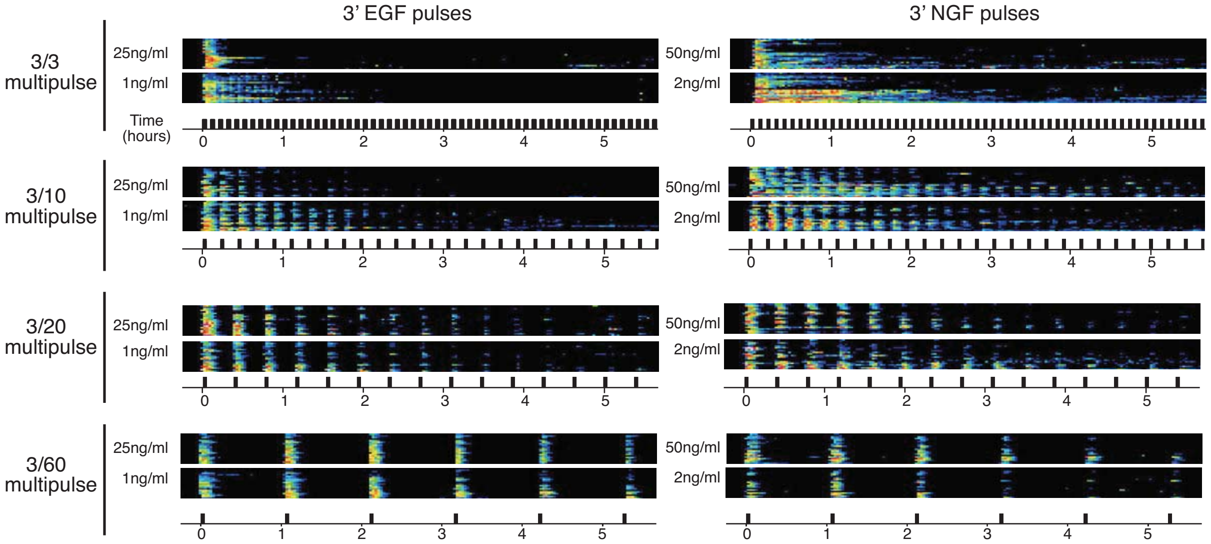

Appendix Figure S 5 Representative single cell ERK activity trajectories in response to 6-hours multipulse 3' GF stimulation regimes at different frequencies.

Waterfall plots of cell-averaged ERs are color coded (n= at least 30 cells for each experiment). GF pulse application is indicated by black bars. GF identity and concentration are also shown.

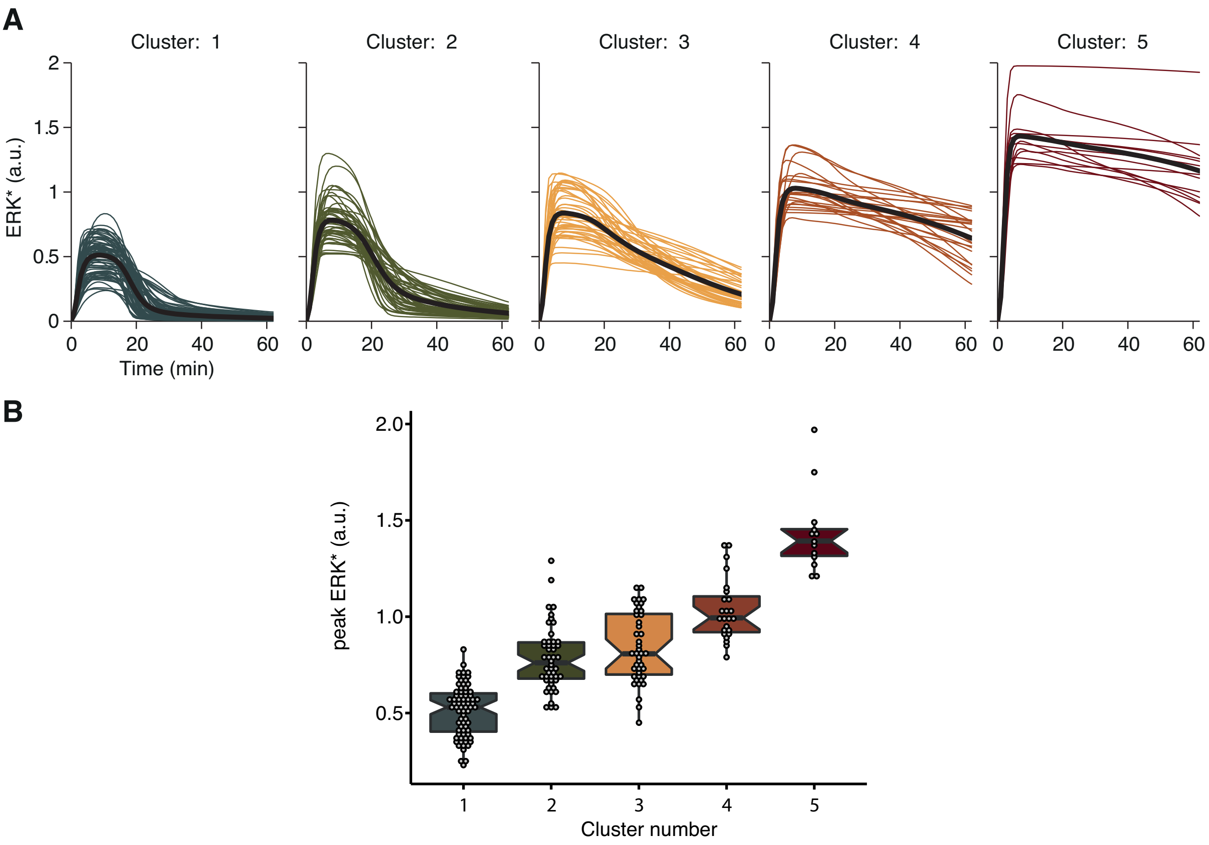

Appendix Figure S6 Quantification of ERK activity trajectories heterogeneity in response to a ensemble-modeled 10’ NGF pulse.

A. Representative ERK activity trajectories identified using k-means clustering. Clusters were sorted according to the mean value of the response within the cluster at 62’ after stimulation to differentiate between transient (clusters 1-3) and sustained responses (clusters 4 & 5). N = 200 simulated cells.

B. Values of response peaks in the 0-10’ interval within each cluster are shown in notched boxplots.

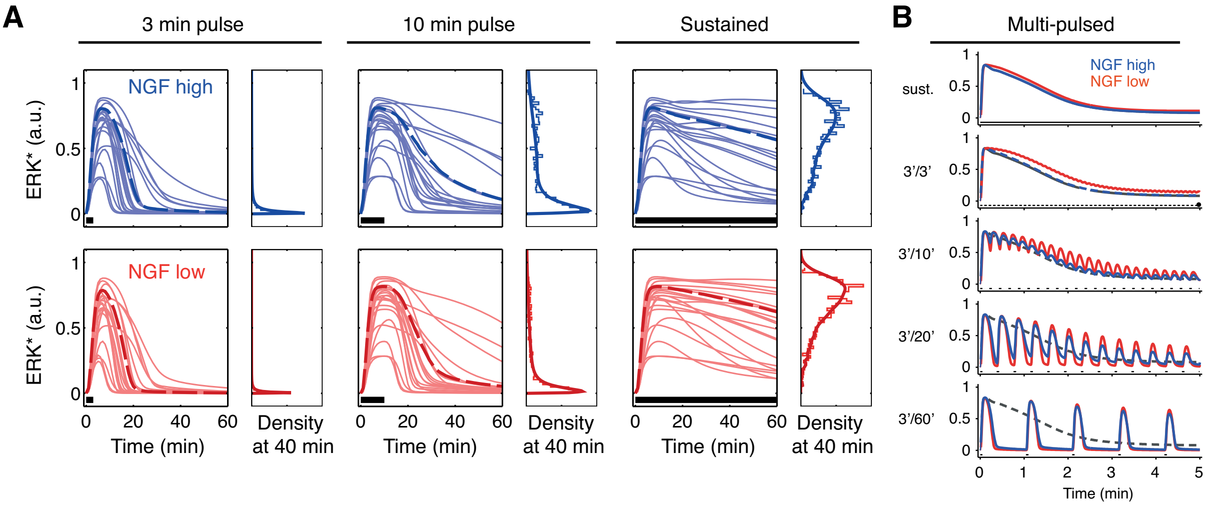

Appendix Figure S7 Simulations of a model variant where the positive feedback depends on receptor activity according to the Hill equation with coefficient 2 and the threshold K50 = 0.25.

A. Semi-deterministic modelling of ERK activation in response to 3' and 10' pulse, as well as a sustained stimulation with low and high NGF. Histograms at 40' are only slightly wider compared to results with linear receptor dependency shown in Fig 4E,F in the main text.

B. Population mean of ERK activation in response to sustained and multi-pulsed low and high NGF stimulation. Dashed line in lower 4 panels corresponds to sustained NGF 50 ng/ml. Time-courses are almost identical compared to results with linear receptor dependency shown in Fig 5A.

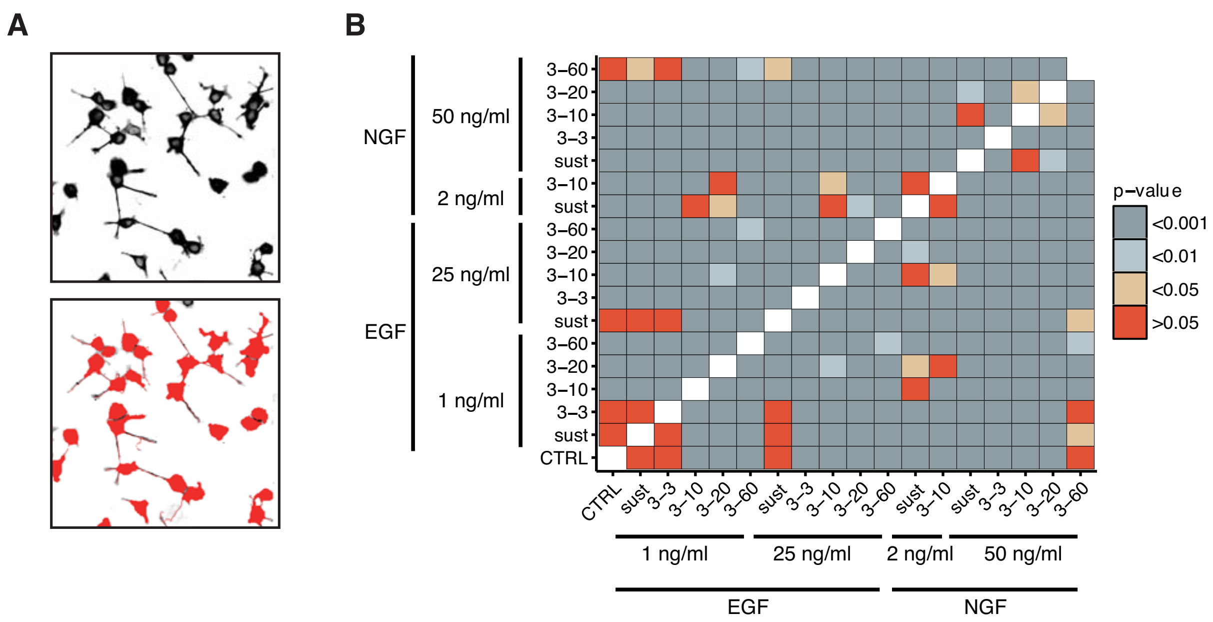

Appendix Figure S8 Additional data for differentiation experiments

A. Example of neurite outgrowth image segmentation. Red outline indicates segmentation of cell somata and neurites.

B. Statistical analysis of neurite outgrowth measurements. The total neurite outgrowth was normalized with respect to cell's major axis length (i.e. the major axis of the ellipse that has the same normalized second central moments as the region). Because of highly skewed distributions, a bootstrapping randomization test was used to evaluate the significance of the observed difference in the mean between conditions (Good, 2006). For every pair of conditions, we resampled the observations 10,000 times and estimated the p-value as the fraction of occurrences for which the difference in the mean was at least as large as the observed difference. P-values were color-coded as indicated in the scheme. Note red color for statistically non-significant measurement pairs.

Appendix Table S1

#	Reaction	Forward rate	Reverse rate	Parameter values	Model version[1]				
					1	2	3	4	5
i)Receptor activation									
1	$R \underset{Ptase1}{\overset{u(t)}{\rightleftharpoons}} R^*$	$k1_R\ R\ u(t)$	$kd1_R\ Ptase_R\ R^*$	$k1_R = 0.5\ min^{-1}$<br>$kd1_R = 0.5\ min^{-1}$<br>$Ptase_R = 1$	x	x	x	x	x
1a	$\emptyset \rightharpoonup R$	k_{syn}	–	$k_{syn} = 0.0014\ min^{-1}$	-	-	-	-	x
1b	$R \rightharpoonup \emptyset$	$k_{deg}\ R$	–	$k_{deg} = 0.0014\ min^{-1}$	-	-	-	-	x
1b	$R^* \rightharpoonup \emptyset$	$k_{deg}\ R^*$	–		-	-	-	-	x
1d	$R^* \rightleftharpoons Ri$	$k_i\ R^*$	$kd_i\ Ri$	$k_i = 0.22\ min^{-1}$<br>$kd_i = 0.044\ min^{-1}$	-	-	-	-	x
1e	$Ri \rightharpoonup \emptyset$	$k_{deg,i}\ Ri$	–	$k_{deg,i} = 0.462\ min^{-1}$	-	-	-	-	x
ii) Ras activation									
6	$Ras \underset{GAP}{\overset{R*}{\rightleftharpoons}} Ras^*$	$k6_R\ R^* \frac{Ras}{K6 + Ras}$	$kd6\ GAP \frac{Ras*}{D6 + Ras*}$	$k6R = 40\ min^{-1}$, $K6 = 1$,<br>$kd6 = 7.5\ min^{-1}$, $D6 = 1$<br>$GAP = 1$	x	x	x	x	x
iii) Raf activation and MAPK cascade									
5	$Raf \underset{Ptase3}{\overset{Ras*}{\rightleftharpoons}} Raf^*$	$k5\ Ras * \frac{Raf}{K5 + Raf} \frac{K_{NFB}^2}{K_{NFB}^2 + NFB*^2}$	$kd5\ Ptase_{Raf} \frac{Raf*}{D5 + Raf*}$	$k5 = 10\ min^{-1}$, $K5 = 1$,<br>$kd5 = 3.75\ min^{-1}$, $D5 = 1$,<br>$K_{NFB} = 0.05$, $Ptase_{Raf} = 1$	x	x	x	x	x
5a	$Raf \xrightarrow{PFB*} Raf^*$	$k_{PFB}\ PFB * \frac{Raf}{K_{PFB} + Raf}$	–	$k_{PFB} = 0\ min^{-1}$<br>(for EGF, see note [2])<br>$k_{PFB} = 0.75\ min^{-1}$ (for NGF)<br>$K_{PFB} = 0.01$	x	x	-	x	x
5b	$Raf \xrightarrow{ERK*} Raf^*$	$k_{PFB}\ ERK * \frac{Raf}{K_{PFB} + Raf}$	–	$k_{PFB} = 0\ min^{-1}$ (for EGF)<br>$k_{PFB} = 0.75\ min^{-1}$ (for NGF)<br>$K_{PFB} = 0.05$	-	-	x	-	-
4	$MEK \underset{Ptase4}{\overset{Raf*}{\rightleftharpoons}} MEK^*$	$k4\ Raf * \frac{MEK}{K4 + MEK}$	$kd4\ Ptase_{MEK} \frac{MEK*}{D4 + MEK}$	$k4 = 2\ min^{-1}$, $K4 = 1$,<br>$kd4 = 0.5\ min^{-1}$, $D4 = 1$<br>$Ptase_{MEK} = 1$	x	x	x	x	x
2	$ERK \underset{DUSP}{\overset{MEK*}{\rightleftharpoons}} ERK^*$	$k2\ MEK * \frac{ERK}{K2 + ERK}$	$kd2\ DUSP \frac{ERK*}{D2 + ERK*}$	$k2 = 2\ min^{-1}$, $K2 = 1$,<br>$kd2 = 0.25\ min^{-1}$, $D2 = 0.1$	x	x	x	x	x
iv) Fast negative feedback									
3a	$NFB \underset{Ptase5}{\overset{ERK*}{\rightleftharpoons}} NFB^*$	$k3\_F\ ERK^* \frac{NFB}{K3 + NFB} \frac{R*^2}{K3R^2 + R*^2}$	$kd3\ Ptase_{NFB} \frac{NFB*}{D3 + NFB*}$	$k3\_F = 0.0286\ min^{-1}$,<br>$K3 = 0.01$, $K3R = 0.85$<br>$kd3 = 0.0057\ min^{-1}$, $D3 = 0.5$<br>$Ptase_{NFB} = 1$	x	-	-	x	-
3b	$NFB \underset{Ptase5}{\overset{ERK*}{\rightleftharpoons}} NFB^*$	$k3\_F\ ERK^* \frac{NFB}{K3 + NFB}$	$kd3\ Ptase_{NFB} \frac{NFB*}{D3 + NFB}$	$k3\_F = 0.0286\ min^{-1}$,<br>$K3 = 0.01$,<br>$kd3 = 0.0057\ min^{-1}$, $D3 = 0.5$	-	x	x	-	x
v) Positive feedback									
7a	$PFB \underset{Ptase6}{\overset{ERK*}{\rightleftharpoons}} PFB^*$	$k7\ ERK^*\ R^* \frac{PFB}{K7 + PFB}$	$kd7\ Ptase_{PFB} \frac{PFB*}{D7 + PFB*}$	$k7 = 0.1\ min^{-1}$, $K7 = 0.1$,<br>$kd7 = 0.005\ min^{-1}$, $D7 = 0.1$<br>$Ptase_{PFB} = 1$	x	-	-	x	-
7b	$PFB \underset{Ptase6}{\overset{ERK*}{\rightleftharpoons}} PFB^*$	$k7\ ERK^* \frac{PFB}{K7 + PFB}$	$kd7\ Ptase_{PFB} \frac{PFB*}{D7 + PFB*}$	$k7 = 0.04\ min^{-1}$<br>(for model 2, see note [3]),<br>$k7 = 0.01\ min^{-1}$<br>(for model 3, see note [3])	-	x	x	-	x
vi) DUSP expression and slow negative feedback									
8	$\emptyset \xrightarrow{ERK*} dusp$	$dusp_{basal} \left(1 + dusp_{ind} \frac{ERK*^2}{K_{dusp} + ERK*^2}\right) \frac{\log(2)}{T_{dusp}}$	–	$dusp_{basal} = 1$<br>$dusp_{ind} = 6$<br>$K_{dusp} = 0.1$<br>$T_{dusp} = 90\ min$	x	x	x	x	-
9	$dusp \rightharpoonup \emptyset$	$dusp\ \log(2)/T_{dusp}$	–	$T_{dusp} = 90\ min$	x	x	x	x	-
10	$\emptyset \xrightarrow{dusp} DUSP$	$dusp\ \log(2)/T_{DUSP}$	–	$T_{DUSP} = 90\ min$	x	x	x	x	-
11	$DUSP \rightharpoonup \emptyset$	$DUSP\ \log(2)/T_{DUSP}$	–	$T_{DUSP} = 90\ min$	x	x	x	x	-

[1] An x-mark "x" indicates that this reaction is present in the model corresponding to this column, a dash "-" indicates that this reaction is absent.
1 – full model
2 – no receptor crosstalk to the feedback components
3 – no delay in the positive feedback and no receptor crosstalk
4 – without DUSP induction
5 – with receptor synthesis, internalisation and degradation

[2] Setting k_{PFB} to zero corresponds to removing the positive feedback loop; this is the case for EGF.
[3] The values of k7 should be set such that a single 10 min pulse of NGF results in a heterogonous ERK response. To achieve that and compensate for the loss of the feed-forward crosstalk from the receptor in models 2 and 3, the value of k7 is reduced in these models.

Model 1: Full model

#	Differential equation			Initial condition
1	d/dt R	=	−v1	1
2	d/dt R*	=	+v1	0
3	d/dt Ras	=	−v6	1
4	d/dt Ras*	=	+v6	0
5	d/dt Raf	=	−v5 − v5a	1
6	d/dt Raf*	=	+v5 + v5a	0
7	d/dt MEK	=	−v4	1
8	d/dt MEK*	=	+v4	0
9	d/dt ERK	=	−v2	1
10	d/dt ERK*	=	+v2	0
11	d/dt NFB	=	−v3a	1
12	d/dt NFB*	=	+v3a	0
13	d/dt PFB	=	−v7a	1
14	d/dt PFB*	=	+v7a	0
15	d/dt dusp	=	+v8 − v9	1
16	d/dt DUSP	=	+v10 − v11	1

Model 2: Model without receptor crosstalk

#	Differential equation			Initial condition
1	d/dt R	=	−v1	1
2	d/dt R*	=	+v1	0
3	d/dt Ras	=	−v6	1
4	d/dt Ras*	=	+v6	0
5	d/dt Raf	=	−v5 – v5a	1
6	d/dt Raf*	=	+v5 + v5a	0
7	d/dt MEK	=	−v4	1
8	d/dt MEK*	=	+v4	0
9	d/dt ERK	=	−v2	1
10	d/dt ERK*	=	+v2	0
11	d/dt NFB	=	−v3b	1
12	d/dt NFB*	=	+v3b	0
13	d/dt PFB	=	−v7b	1
14	d/dt PFB*	=	+v7b	0
15	d/dt dusp	=	+v8 − v9	1
16	d/dt DUSP	=	+v10 − v11	1

Model 3: Model with no delay and no receptor crosstalk

#	Differential equation			Initial condition
1	d/dt R	=	−v1	1
2	d/dt R*	=	+v1	0
3	d/dt Ras	=	−v6	1
4	d/dt Ras*	=	+v6	0

5	d/dt Raf	=	−v5 – v5b	1
6	d/dt Raf*	=	+v5 + v5b	0
7	d/dt MEK	=	−v4	1
8	d/dt MEK*	=	+v4	0
9	d/dt ERK	=	−v2	1
10	d/dt ERK*	=	+v2	0
11	d/dt NFB	=	−v3b	1
12	d/dt NFB*	=	+v3b	0
13	d/dt dusp	=	+v8 − v9	1
14	d/dt DUSP	=	+v10 − v11	1

Model 4: Model without ERK-induced DUSP expression

#	Differential equation			Initial condition
1	d/dt R	=	−v1	1
2	d/dt R*	=	+v1	0
3	d/dt Ras	=	−v6	1
4	d/dt Ras*	=	+v6	0
5	d/dt Raf	=	−v5 – v5a	1
6	d/dt Raf*	=	+v5 + v5a	0
7	d/dt MEK	=	−v4	1
8	d/dt MEK*	=	+v4	0
9	d/dt ERK	=	−v2	1
10	d/dt ERK*	=	+v2	0
11	d/dt NFB	=	−v3	1
12	d/dt NFB*	=	+v3	0
13	d/dt PFB	=	−v7a	1
14	d/dt PFB*	=	+v7a	0
15	d/dt dusp	=	0	1
16	d/dt DUSP	=	0	1

Model 5: Model including receptor internalization and degradation

#	Differential equation			Initial condition
1	d/dt R	=	+v1a − v1 − v1b	1
2	d/dt R*	=	+v1 − v1d − v1c	0
3	d/dt Ri*	=	+v1d − v1e	0
4	d/dt Ras	=	−v6	1
5	d/dt Ras*	=	+v6	0
6	d/dt Raf	=	−v5 − v5a	1
7	d/dt Raf*	=	+v5 + v5a	0
8	d/dt MEK	=	−v4	1
9	d/dt MEK*	=	+v4	0
10	d/dt ERK	=	−v2	1
11	d/dt ERK*	=	+v2	0
12	d/dt NFB	=	−v3	1
13	d/dt NFB*	=	+v3	0
14	d/dt PFB	=	−v7b	1
15	d/dt PFB*	=	+v7b	0
16	d/dt dusp	=	+v8 − v9	1

17	d/dt DUSP	=	$+v10 - v11$	1

Appendix Note 1

Because the positive feedback was so important for explaining our single cell pulsing data, we analyzed it more detail. The linear dependency of PFB on R* (Appendix Table I, equation 7a) is not critical, but was the simplest way of modeling this dependency. Albeit there are some minor quantitative differences when a Hill-shaped kinetic is used, model-simulated ERK* trajectories (Appendix Figure 2). Most importantly, the distribution of the time-course responses for the 3 and 10 min NGF pulses for low and high dosages are virtually identical to the main results where linear kinetics is used (compare Appendix Figure 7A and Fig 5E,F). This also holds true for multipulse datasets (compare Appendix Figure 7B and Fig 6A).

To perform the above-mentioned analysis, we used the following equations.

Main results (linear dependency on receptor activation):

$$V7a = k7\ ERK^*\ \ R^*\ \frac{PFB}{K7 + PFB}$$

Supplementary results (Hill dependency with coefficient *2*):

$$V7a = k7\ ERK^*\ \frac{(R^*)^2}{0.25^2 + (R^*)^2}\ \frac{PFB}{K7 + PFB}$$

In these equations for the positive feedback, the actual parameter values are more important than the form of the equation. The feedback has to be activated at low levels of receptor activity in order to explain our experimental data, which can be achieved by using a linear dependency, or a Hill-shaped dependency with relatively low values for the half-activation parameter K50 << 1 (here K50 = 0.25). For example, using a larger K50 of 0.5 in the above equation did not explain our experimental data because all simulated single cell responses were transient and there were no sustained responses for 10’ NGF at high dosage. Moreover, these subtle parameter differences were not visible for sustained NGF stimulation, thus further highlighting the usefulness of our pulsing experiments. The dependency of the “fast” negative feedback on active receptor is implemented as Hill function with H=2 to facilitate a higher threshold (K3R = 0.85) for activation of the receptor cross-talk.

Appendix References

Good PI (2006) Permutation, Parametric, and Bootstrap Tests of Hypotheses. *Springer Series in Statistics*
