# Supplementary material for: Frequency modulation of ERK activation dynamics rewires cell fate
Source: Mol Syst Biol. 2015 Nov 30;11(11):838. doi: 10.15252/msb.20156458 (PMC4670727; doi:10.15252/msb.20156458)
Supplement: Supplementary file 2 — Expanded View Figures PDF [file MSB-11-838-s002.pdf]

Expanded View Figures

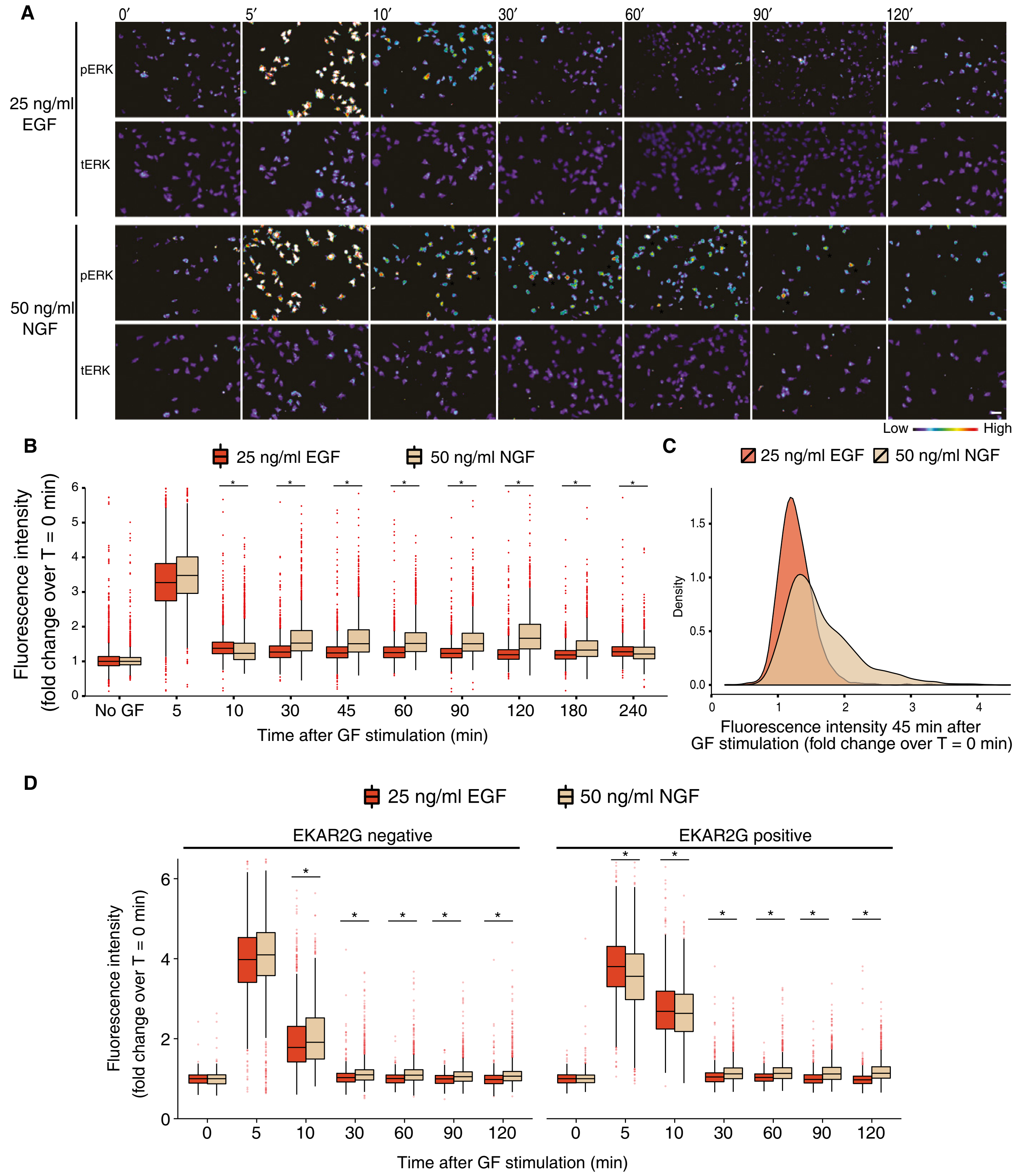

Figure EV1.

Figure EV1. Time course of single-cell ERK activation steady states in response to sustained GF stimulation.

A PC-12 cells in conventional dishes were stimulated with 25 ng/ml EGF or 50 ng/ml NGF, fixed at the indicated time points after stimulation, and stained with anti-phosphoERK and anti-ERK2 antibodies. Images are color-coded for phosphoERK or ERK2 signal intensity. Note that the immunostaining intensity images have been scaled so as to slightly saturate phosphoERK signals at the 5′ time point. This provides the required dynamic range to clearly distinguish heterogeneous activation states within the cell population in response to NGF stimulation at later time points. At time points after the 1st ERK activity peak, red asterisks indicate specific cells that exhibit elevated ERK activity. Scale bar: 50 μm.

B Population distributions of phosphoERK signals. Boxplots of cell-averaged phosphoERK signals for 25 ng/ml EGF and 50 ng/ml NGF dosages, n= at least 1,200 cells/condition/time point; * < 0.05, bootstrapping randomization test for the observed difference in the mean. Median, interquartile range (box), and data within 1.5 IQR range of the lower and upper quartiles (whiskers) are shown. Measurements are normalized to $t = 0'$. DAPI-stained images were segmented in CellProfiler with global Otsu thresholding. Cell bodies were identified by expanding nuclei based on total ERK staining. Whole-cell mean intensity was calculated from phosphoERK channel after correcting images for uneven illumination and after subtracting mean background intensity.

C Density plot of phosphoERK signals distribution at 25 ng/ml EGF and 50 ng/ml NGF at 45′ post-stimulation. Measurements are normalized to $t = 0'$.

D Population distributions of phoshoERK signals in native PC-12 cells or PC-12 cells expressing the EKAR2G biosensor. PC-12 cells expressing or not expressing EKAR2G were mixed, stimulated with either 25 ng/ml EGF or 50 ng/ml NGF, fixed at the indicated time points, and immunostained using anti-phosphoERK antibodies. PC-12 cells expressing or not expressing EKAR2G were identified using the FRET channel and analyzed as in (B), n= at least 990 cells (* < 0.05, two sample t-test for the mean). Note that 1st ERK activity peak desensitizes slightly more slowly in PC-12 cells expressing EKAR2G versus native PC-12 cells. However, increased phosphoERK signals are still observed when NGF- and EGF-treated cells are compared at time points after 30′.

 © 2015 The Authors

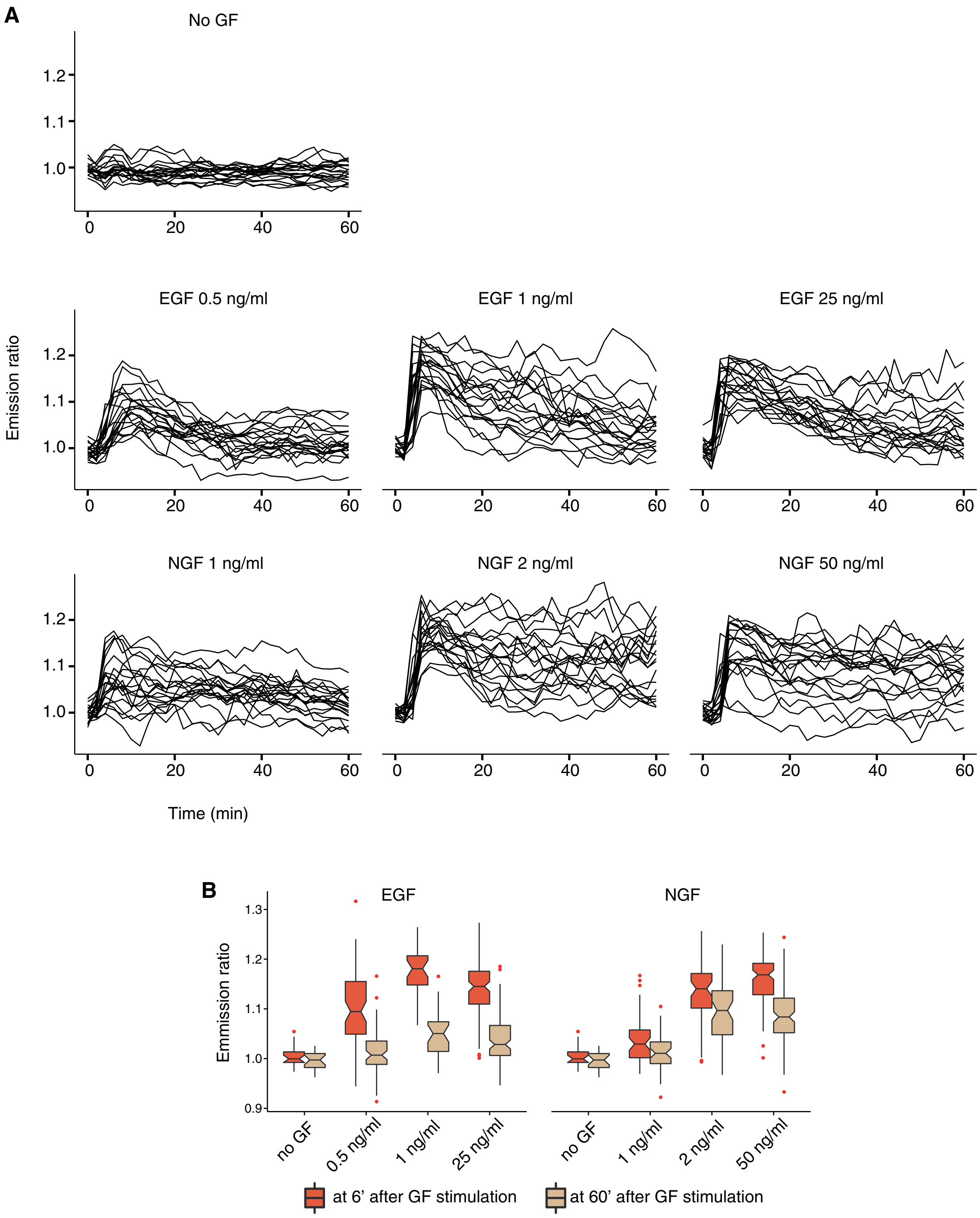

Figure EV2. Threshold GF concentrations required for robust ERK activation.

ERK activation dynamics were evaluated in the microfluidic device at different EGF and NGF concentrations in response to sustained GF stimulation.

A Representative ERK activity trajectories in response to different GF dosages (*n* = 20).

B Distribution of cell-averaged ERs at 6′ (maximum of 1st ERK activity peak), or at 60′ after GF stimulation (long-term ERK activity). Notched boxplots with median, interquartile (box), and 1.5 IQR (whiskers) range are shown for at least *n* = 30 cells. Note ERK activity adaptation for EGF versus sustained ERK activity for NGF at the 60′ time point.

© 2015 The Authors

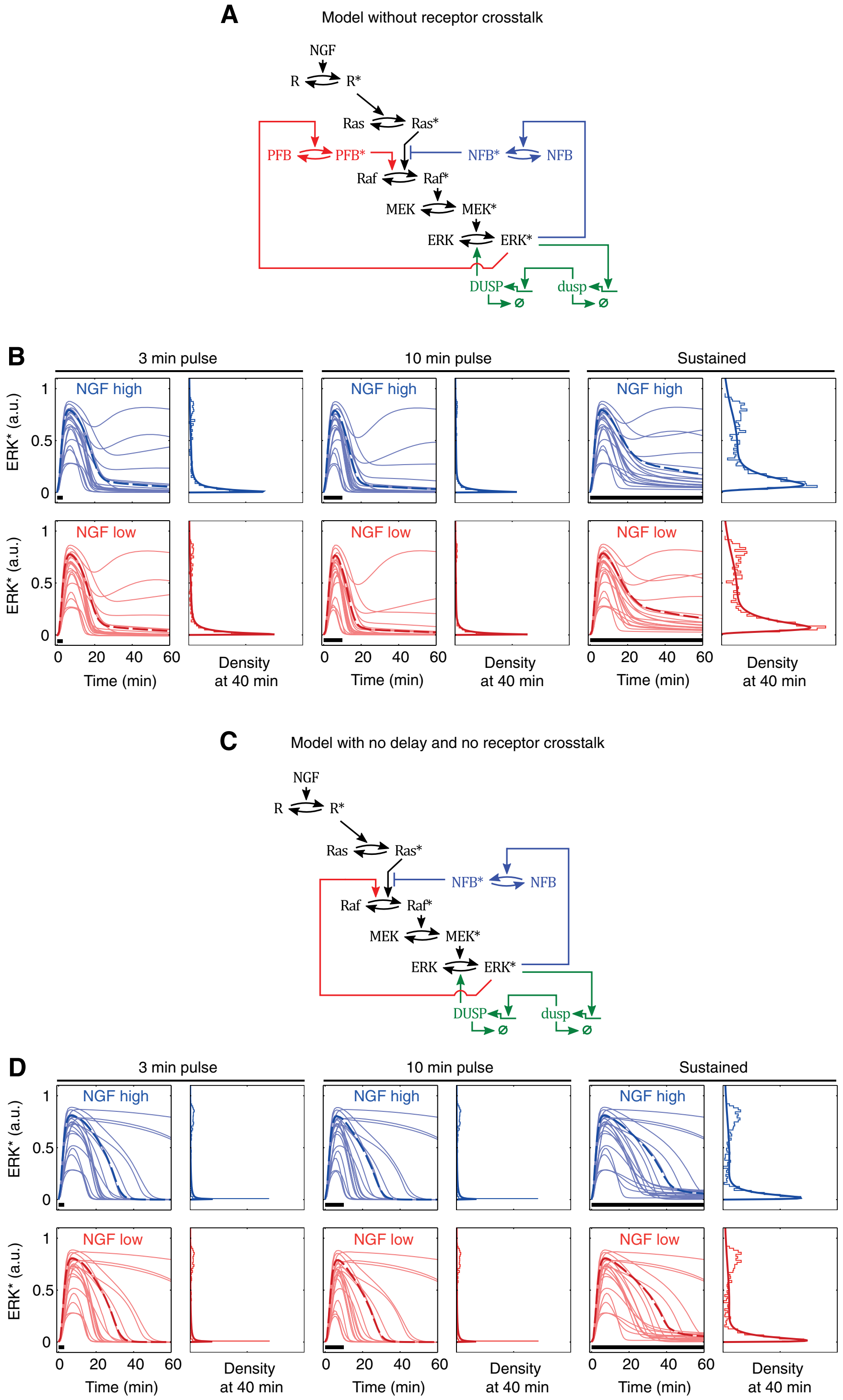

Figure EV3. Semi-deterministic modeling of pulsed GF stimulation using alternate models.
Ensemble modeling of 3′ or 10′ EGF and NGF pulses with variant models was performed. An ordinary differential equation (ODE) model was run 1,000 times with total signaling component concentrations drawn from the log-normal distribution shown in Fig 5C. Model parameters are the same as in the full model.

- A Scheme of a model variant without receptor crosstalk.
- B Simulated ERK* trajectories in response to the indicated NGF stimulation schemes according to variant model shown in (A).
- C Scheme of a model variant with no delay and no receptor crosstalk.
- D Simulated ERK* trajectories in response to the indicated NGF stimulation schemes according to variant model shown in (C).

 © 2015 The Authors

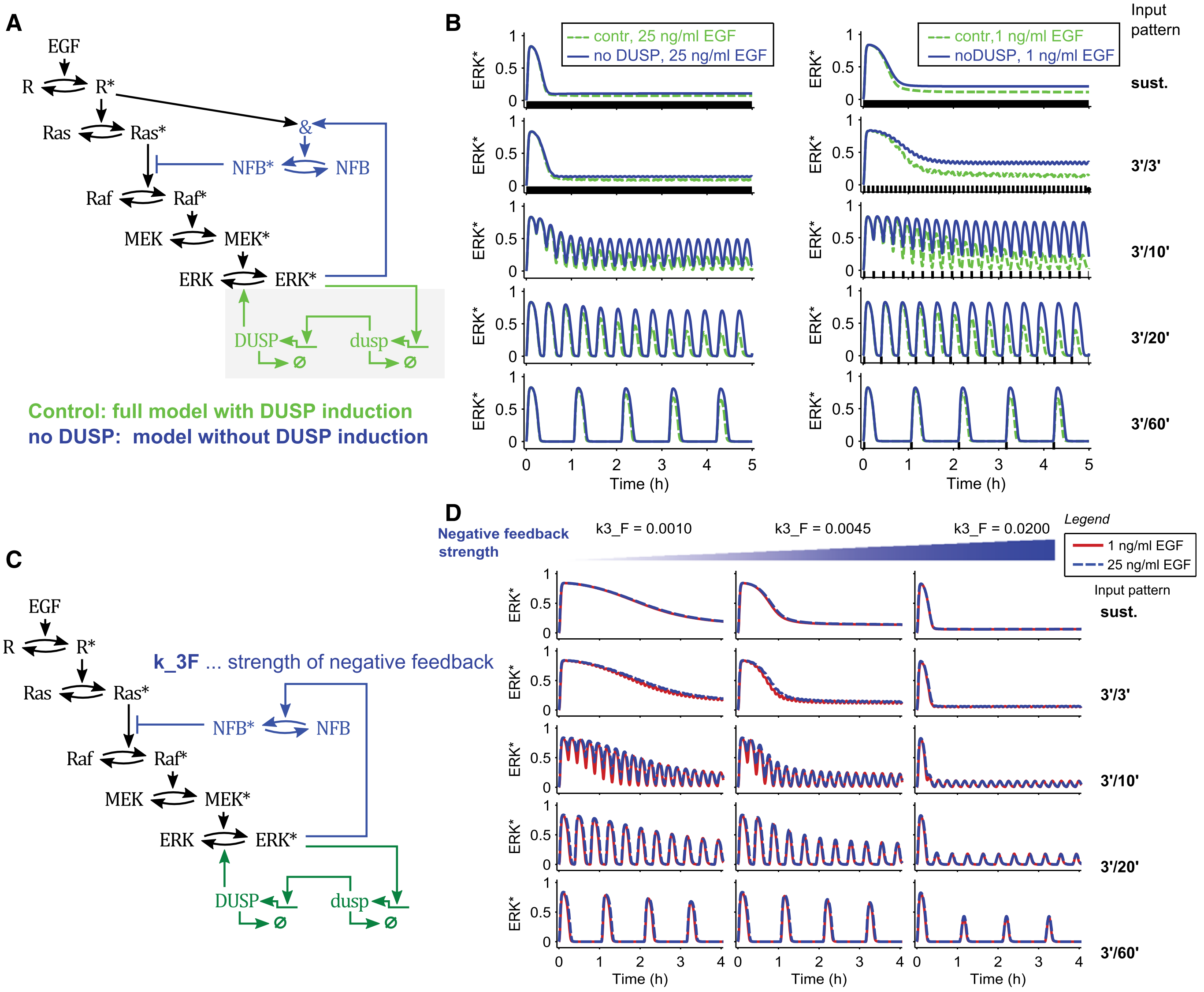

Figure EV4. Deterministic modeling of multi-pulse-induced ERK activation dynamics using alternate models.

A Scheme of a model variant in which the DUSP module has been removed.

B Simulated ERK* trajectories showing a comparison of the full model (control, green) with the model lacking DUSP induction (no DUSP, blue). Results for high (left panels) and low (right panels) EGF dosages are shown. Rows correspond to different stimulation patterns: sustained GF stimulation, 3′/3′, 3′/10′, 3′/20′, and 3′/60′ multi-pulse regimes. Much lower long-term decay is observed in the model in which the DUSP module was removed in the multi-pulse experiments. Parameters of the model with and without DUSP induction are the same as in the full model.

C Scheme of a model variant in which the feed-forward crosstalk to the ERK-Raf negative feedback loop has been removed.

D Simulated ERK* trajectories. Rows correspond to different stimulation patterns: sustained GF stimulation, 3′/3′, 3′/10′, 3′/20′, and 3′/60′ multi-pulse regimes. No dose-dependent differences are observed. Columns correspond to different parameter values for the negative feedback strength, demonstrating that this result is robust. All parameters, except k3_F, are the same as in the full model.

© 2015 The Authors

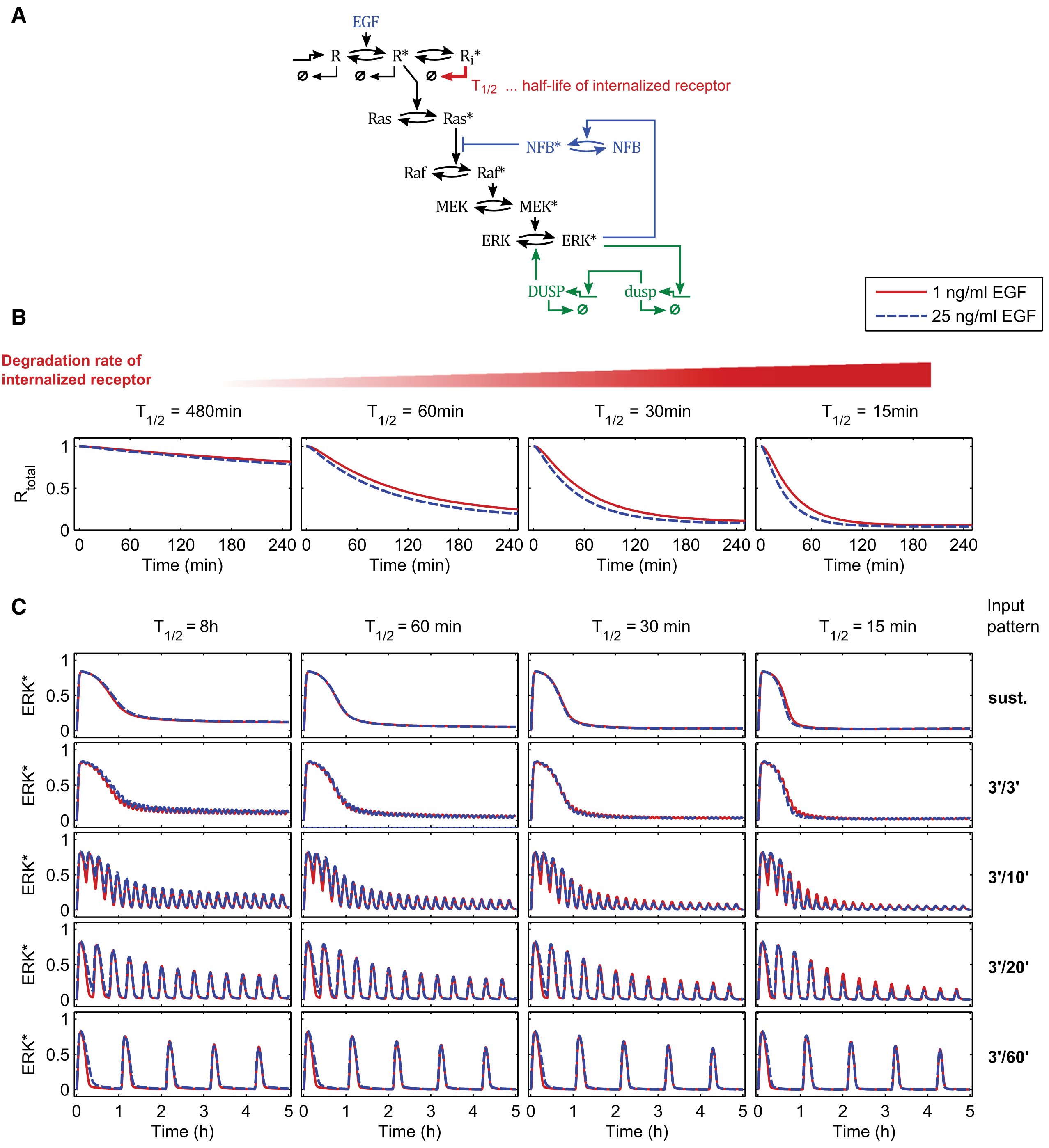

Figure EV5. Deterministic modeling of multi-pulse-induced ERK activation dynamics using alternate models—model variant that features receptor internalization and degradation instead of a feed-forward loop onto the negative feedback.

A Scheme of the alternative model. The receptor is synthesized and degraded with a half-life of 8 h. After activation by binding EGF, the receptor is internalized (0.22 min^{-1}) and recycled (reverse rate is 20% of the forward rate). The internalized receptor is degraded much faster than the non-internalized receptor (indicated by $T_{1/2}$). All parameters, except $T_{1/2}$, are the same as in the full model.

B Simulated changes in the total receptor levels $R_{total} = R + R^* + R_i^*$ in response to constant stimulation with EGF for different parameter values for the half-life of the internalized receptor $T_{1/2}$.

C Simulated ERK* trajectories in response to different stimulation patterns. From top to bottom: sustained GF stimulation, 3′/3′, 3′/10′, 3′/20′, and 3′/60′ multi-pulse regimes. No notable dose-dependent differences are observed. Columns correspond to different parameter values for the half-life of the internalized receptor $T_{1/2}$, demonstrating that this result is robust.

 © 2015 The Authors
