# Supplementary material for: Frequency modulation of ERK activation dynamics rewires cell fate
Source: Mol Syst Biol. 2015 Nov 30;11(11):838. doi: 10.15252/msb.20156458 (PMC4670727; doi:10.15252/msb.20156458)
Supplement: Supplementary file 8 — Source Data for Figure 1 [file MSB-11-838-s007.zip › SourceData Fig 1/SourceData_Figure_1D/README.rtf]

Source Data for Figure 1D. Uncropped gels.
